# Effectiveness of inulin-type on the iron bioavailability in anemic female rats fed bio-yogurt

**DOI:** 10.1039/d0ra08873k

**Published:** 2021-01-06

**Authors:** Osama Mohammed, Noha Dyab, Ehab Kheadr, Nassra Dabour

**Affiliations:** Department of Zoology, College of Science, King Saud University Riyadh 11451 Saudi Arabia; Functional Foods and Nutraceuticals Laboratory (FFNL), Department of Dairy Science and Technology, Faculty of Agriculture, University of Alexandria 21545 Alexandria Egypt nassradabour@ymail.com +20-35922780 +20-35921960 +20-35921862 +20-35915427

## Abstract

It is well-documented that iron deficiency leads to anemia, which is the utmost critical problem of nutrition worldwide. Inulin, indigestible polysaccharides, or prebiotic agents may act as vehicles to enhance the iron bioavailability through the formation of the polysaccharide–iron complex. The present study was undertaken to evaluate the therapeutic effects of yogurt fortified with iron and supplemented by long- or short-chain inulin on the growth status, blood parameters, antioxidant capacity, and liver function enzymes in anemic rats. Five animal groups were assigned as the control (G1), which were fed a standard diet and there were four anemic groups, in which haemolytic anemia was induced by phenylhydrazine. The anemic rats were divided into 4 groups according to the regime of feeding as G2: control anemic group fed low-iron diet while the remaining anemic groups were fed yogurt fortified with Fe_2_(SO_4_)_3_ without inulin (G3) or with either long- (G4) or short-chain (G5) inulin. The results showed that the animals subjected to treatment G4 had the highest (*P* ≤ 0.05) weight gain and organ coefficient compared with other anemic groups (G2, G3, and G5). Among the anemic groups, the animals that belonged to G4 showed a significant restorative effect by returning the hemoglobin and hematocrit levels and the red blood cell count to the normal control liver. Also, the liver iron content, enzymatic activities, and antioxidant capacities improved in the animals subjected to G4 and G5 treatment groups. The histological structures of the liver tissues of the animals that belonged to G4 and G5 were extremely close to that of the normal control liver. Long-chain inulin-containing yogurt exhibited the best effects in terms of iron supplementation, bioavailability, and antioxidant activities. This formula might be a potential new iron supplement and a good functional food candidate.

## Abstract 2

It is well-documented that iron deficiency leads to anemia, which is the utmost critical problem of nutrition worldwide.

## Graphical abstract

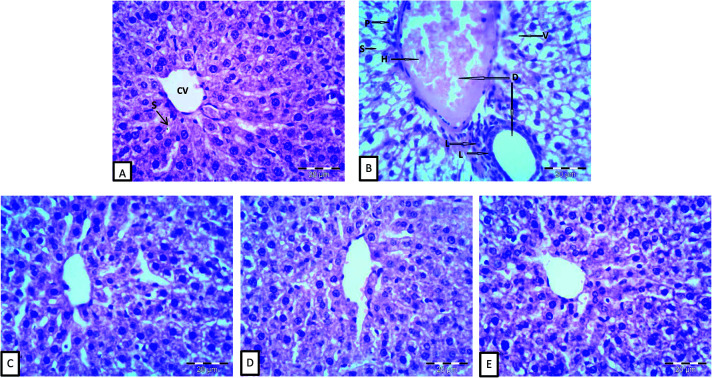

## 1 Introduction

Iron is an essential element for life as it is considered to be an important component of human hemoglobin, cytochrome enzymes, and many reductases.^1^ Iron has an important role in oxygen transportation, deoxyribonucleic acid synthesis, and energy metabolism.^2^ Iron deficiency is the most prevalent nutrient deficiency worldwide and has been considered to be the common cause of anemia.^3,4^ Iron deficiency anemia (IDA) has been reported to adversely affect mental and physical development.^5^ Also, IDA can lead to an increment in lipid peroxidation and reduction in antioxidant defenses,^6^ immune function, and nervous system disorders,^7,8^ and it also causes emaciation.^9,10^

IDA's prevalence is mostly common among children and women, particularly in developing countries.^11^ Iron deficiency during pregnancy has been shown to be associated with prematurity, perinatal mortality,^12^ poor foetal growth, and survival, and a high risk of chronic disease in newborns.^13^ Anemia caused by iron deficiency is generally characterized by a low level of blood haemoglobin, which can delay normal infant motor and mental functions and immunity.^14^

The most common method for preventing IDA is enough iron supplementation.^15^ In practice, iron salts (*e.g.*, Fe_2_(SO_4_)_3_) are commonly used to compensate for iron deficiency as oral supplements.^16,17^ However, the long-term use of oral iron supplements has been shown to cause many gastrointestinal disorders including, nausea, vomiting, abdominal pain, and constipation.^18–20^ Therefore, it would be better to develop functional foods to circumvent the prevalence of anemia and improve the iron bioavailability. Among functional foods, prebiotic containing-products are believed to be efficient in improving the iron uptake due to the formation of the prebiotic–iron complex, which can effectively enhance the iron bioavailability. This concept was developed since food supplementation with iron salts has proved to be less efficient in delivering the metal to the human body.^21,22^ On the other hand, polysaccharides are widely used in the formation of many foods as the gelling material and/or prebiotic agents. Thus, polysaccharides from different sources, *e.g.*, soybean, malt, and chitosan, have been evaluated for their ability to form polysaccharide-iron complexes.^23–25^ These complexes have exceptional water solubility, iron-binding ability, and do not cause gastrointestinal irritation at oral doses.

Inulin, a fructan polysaccharide, is widely used in the food industry as a texture modifier, fat replacer, or low-calorie sweetener. It is found in large quantities in Jerusalem artichoke, chicory root, garlic, asparagus root, and dandelion root.^26^ Inulin has potential therapeutic effects such as improved gut microbiota, increased mineral absorption, stimulation of immune functions, reduced risks of irritable bowel diseases and constipation, and has a positive effect against diabetes.^27^ The polymerization degree (DP) of inulin ranges from 2 to 60 units. The DP value has an important consideration as it may affect the inulin functionality and therapeutic effects. A greater comprehension of these effects would be important for designing functional foods with enhanced iron bioavailability. Consequently, the formation of the inulin/iron complex has been proposed to improve the bioavailability of iron. Guggi *et al.*^28^ proposed the use of inulin derivatives (mainly thiolated/carboxylated inulin) to complex iron and to improve the interaction of inulin with the intestinal mucosa. This interaction would increase the residence time of the inulin–iron complex in the gastro-intestinal tract and promote the absorption of iron. Also, inulin has potential biocompatibility and is biodegradable in the intestinal tract. Also, inulin has the ability to form complexes with iron assured the presence of the Fe iii in a more available from Pitarresi *et al.*^29^ Inulin and oligofructose prebiotics have been shown to improve the intestinal absorption of iron in rats with anemiaIDA.^30^

Our previous study indicated that the inclusion of 1% inulin (either short-chain or long-chain) or 40 mg L^−1^ of iron salts (either ferric chloride or ferric sulfate) and/or their combinations in milk did not significantly disturb the yogurt fermentation process and yogurt sensorial attributes.^31^ The study also showed that the *in vitro* bioavailability of calcium and iron was higher in the case of yogurt-fortified with ferric sulfate compared to that in ferric chloride. To date, there have been no *in vivo* studies about the effect of inulin on the iron bioavailability. In this context, the present study aimed to develop bio-yogurt fortified with iron and supplemented with short- or long-chain inulin to improve the bio-availability of iron in anemic rats, and thoroughly investigates the biochemical characteristics and liver histopathological futures. Indeed, this present study reflects the art of functional food formulation *via* inexpensive, convenient, and effective means.

## 2 Materials and methods

### 2.1 Yogurt manufacturing

Four low-fat yogurt batches were made as previously described by Dabour *et al.*^31^ Partially skimmed fresh cow's milk (1.7% fat and 10.5% total solids), obtained from the pilot farm at Alexandria University, was heated at 45 °C and divided into 4 lots as follows.

Batch A (inulin free) was fortified with 90 mg kg^−1^ Fe_2_(SO_4_)_3_ and 3.5% skimmed milk powder.

Batch B (inulin free) was fortified with 10 mg kg^−1^ Fe_2_(SO_4_)_3_ and 3.5% skimmed milk powder.

Batch C was fortified with 90 mg kg^−1^ Fe_2_(SO_4_)_3_, 1.5% skimmed milk powder, and 4% long-chain inulin (degree of polymerization (DP) > 23 fructose units were from Sensus Company, Roosendaal, Netherlands).

Batch D was fortified with 90 mg kg^−1^ Fe_2_(SO_4_)_3_, 1.5% skimmed milk powder, and 4% short-chain inulin (DP of 8 obtained from Sensus Company).

Both short- and long-chain inulins were extracted from chicory and composed of a mixture of linear fructose polymers with a terminal glucose unit, coupled through β(2–1) bonds.

Following fortification, all the batches were heated at 95 °C for 5 min, cooled to 42 °C, inoculated (0.01%, w/w) with direct set yogurt starter culture (YO-Flex®, Chr. Hansen's Lab., Denmark), filled into 100 mL plastic cups, and incubated at 42 °C until the pH reached 4.6. The cups were then kept in a refrigerator (6 ± 1 °C) for a maximum storage period of 7 days.

### 2.2 Animals

Forty female albino rats (ten-week-old, 140–160 g) were obtained from the animal house of the Faculty of Agriculture, Alexandria University. The animals were kept at 25 ± 1 °C with 12 h dark and light cycles and the room humidity was about 50 ± 5%.^32^ The iron-free standard diet was formulated according to Reeves *et al.*,^33^ as shown in Table 1. Diet and tap water were provided *ad libitum*. After 2 weeks of acclimatization, the animals were randomly distributed into different groups (8 animals per group), which were assigned as G1 to G5, as described below.

**Table 1:** Formulation of iron-free standard diet

Diet ingredients	(%)
Whey proteins concentrate (80%)	20.0
Corn oil	10.0
Corn starch	52.5
Sucrose	10.0
Fe-free mineral mixture	3.5
Vitamin mixture	1.0

### 2.3 Induction of anemia and the experimental design

The experimental protocol was approved by the Institutional Animal Care and Use Committee (IACUC) under permission no. (AU08190625334). The experiment was conducted in animal facilities at the Faculty of Agriculture, Alexandria University. The experimental procedures followed the guidelines issued by the National Research Council.^34^

Animals belonging to groups 2 to 5 were subjected to treatment with phenylhydrazine (Sigma Aldrich Company, St. Louis, MO, USA) to induce haemolytic anemia, according to the protocol described by Moreau *et al.*^35^ Briefly, phenylhydrazine was dissolved in sterilized saline and injected into the animal body at 40 mg kg^−1^ body weight through the peritoneal route. The injection was repeated twice in 24 h following the first injection at 9 h intervals between the second and third injection. Fifteen hours after the third injection, blood samples were withdrawn and subjected to haemoglobin determination. The results revealed that the levels of blood hemoglobin were 13.40 ± 0.23 and 8.00 ± 0.89 g dL^−1^ for healthy and anemic rats, respectively.

The animal groups assigned in the present study were as follows:

G1: non-anemic animals fed with an iron-free standard diet mixed with (1 : 1) inulin-free yogurt containing 90 mg kg^−1^ Fe_2_(SO_4_)_3_.

G2: anemic animals fed with an iron-free standard diet mixed with (1 : 1) inulin-free yogurt containing 10 mg kg^−1^ Fe_2_(SO_4_)_3_.

G3: anemic animals fed with an iron-free standard diet mixed with (1 : 1) inulin-free yogurt containing 90 mg kg^−1^ Fe_2_(SO_4_)_3_.

G4: anemic animals fed with an iron-free standard diet mixed with (1 : 1) yogurt containing 90 mg kg^−1^ Fe_2_(SO_4_)_3_ and 4% long-chain inulin.

G5: anemic animals fed with an iron-free standard diet mixed with (1 : 1) yogurt containing 90 mg kg^−1^ Fe_2_(SO_4_)_3_ and 4% short-chain inulin.

Following the formulation of different diets, samples were taken to determine the iron content by ashing the samples at 550 °C, followed by measurements with an atomic absorption spectrometer (Thermo Scientific, Cambridge, United Kingdom). The iron concentration in the diets presented to the animals subjected to treatments G1, G3, G4, and G5 was 45.30 ± 0.20 mg kg^−1^ while animals in group G2 received a diet containing 5.10 ± 0.05 mg Fe per kg (by analysis). The iron concentrations of the standard and anemic diets were reported to be 45 and 5 mg kg^−1^, respectively.^33,36^ The experiment lasted 4 weeks.

At the end of the experiment, the animals were fasted overnight and lightly anesthetized with diethyl ether. The rats were sacrificed and the organs (liver, kidney, brain, lung, heart, and testis) were immediately removed, washed twice with saline solution, dried using tissue paper, weighed, and stored at −80 °C until used. Fresh liver tissues were subjected to histological analysis directly after they had been excised from the animals.

### 2.4 Body and organs' weight

The body weight gain of each animal was determined weekly and the results are presented as the total weight gained after 4 weeks of the experiment, as described by El-Saadany and Abd-Elhaleem^37^ using the following equation:Body weight gain = *W*_f_ − *W*_0_,where *W*_f_ and *W*_0_ are the final and initial weights recorded at the end and the beginning of the experiment, respectively.

### 2.5 Organ coefficients

The liver, spleen, heart, and kidney were separated from the dissected rats. The organs were washed with a sterilized physiological solution to remove the blood residual, dried with a tissue paper, and then weighed. The organ coefficients were calculated by the following equation according to Liu *et al.*^38^Organ coefficient (g/100 g) = [organ weight (g)/rat body weight (g)] × 100

### 2.6 Hematological parameters

Blood was collected from the major blood artery. All the hematological parameters were measured by a hematology analyzer Sysmex K-1000D (Sysmex, Tokyo, Japan).

### 2.7 Sera preparation

Blood samples were collected in plastic tubes and placed at room temperature for 30 min. Serum was obtained by the centrifugation of the samples at 3000 rpm for 20 min at 4 °C (Hettich Universal 32R, Germany). The sera were pipetted, kept in a 1.5 mL microtube, and stored at −80 °C until the assays of biochemical parameters and enzyme activities.

### 2.8 Liver antioxidant markers

After excision, the livers were weighed, immediately washed using chilled saline solution, dried with tissue paper, and stored at −80 °C until the homogenization process. Frozen livers were minced and homogenized (10% w/v) in ice-cold sucrose buffer (0.25 M to make a 1 : 10 suspension) in a Wise Tis® HG-15D Homogenizer (Daihan-Scientific, India). The homogenate was centrifuged at 10 000 rpm for 30 min at 4 °C. The resultant supernatant was then stored at −80 °C. Lipid peroxidation in the supernatant of the liver homogenate was measured as thiobarbituric acid-reactive substances (TBARS), according to the method of Tappel and Zalkin.^39^ The activities of superoxide dismutase (SOD) and glutathione peroxidase (GPx) in the supernatant were determined by the methods previously described by Misra and Fridovich^40^ as well as Chiu *et al.*,^41^ respectively.

### 2.9 Liver function

The activity of alkaline phosphatase (ALP) in the serum was assayed by a commercial kit (Bio Systems S.A Costa Brava 30, Barcelona, Spain) according to the method of the International Federation of Clinical Chemistry.^42^ Aspartate aminotransferase (AST) and alanine aminotransferase (ALT) activities in the plasma were determined by the colorimetric method, as reported by Reitman and Frankel.^43^ Total plasma protein was quantified using biuret reagent.^44^

### 2.10 Liver histology

The liver tissues were excised and fixed in neutral buffered formalin 10% for 14–18 h and then dehydrated through ascending grades of ethyl alcohol until they reached the absolute alcohol level (1 h). The fixed tissues were sectioned at 4 to 5 mm thickness. The tissue sections were collected on glass slides, deparaffinized, and stained with hematoxylin and eosin stain.^45^ The sections were then examined and observed under a light microscope at 400× magnification.

### 2.11 Statistical analysis

Statistical analysis was performed using the SPSS 25.0 software (Statistical Package for Social Sciences, USA). Analysis of variance (ANOVA) of the data was conducted and the means for the property values were separated (*P* ≤ 0.05) with Student–Newman–Keuls (SNK) and Duncan multiple range tests. The differences were considered significant at *P* ≤ 0.05.

## 3 Results and discussion

### 3.1 Body weight gain

Once the anemic status was induced and established in the animals, they became less active and had a faster breathing rate compared to that of the healthy ones (G1). During the experiment, symptoms of iron deficiency were evident among anemic animals as their skin, ears, claws, tails, eyes, and noses gradually became pale, and the fur was sparse and rough. These characters became worse in G2 animals with the progress of the experimental time due to the lack of iron intake but were less evident with iron supplementation and inulin intake (G3, G4, and G5). The present results were in agreement with those reported by He *et al.*^46^ and Zhang *et al.*^47^ for anemic animals fed with the oligosaccharide–iron complex.

As shown in Table 2, the body weight gain of the rats in the anemic groups exhibited significant differences (*P* ≤ 0.05) compared with the control group. The lowest (*P* ≤ 0.05) body weight gain was reported in the anemic group (G2), which was fed with the low-iron diet. This result confirmed the establishment of the anemic model and was corresponding with the study performed by Zhang *et al.*^47^ and Wang *et al.*^48^ After iron and inulin supplementation, the body weight of the anemic rats significantly increased (*P* ≤ 0.05) over that of the animals subjected to the G2 group. At the end of the experiment, the highest body weight gain was exhibited in G5, which is the group containing anemic animals fed a standard diet mixed with yogurt containing 4% short-chain inulin and 90 mg iron. Also, rats fed yogurt-containing long-chain inulin (G4) gained body weight very close to that of the non-anemic group control (G1). This implied that our feeding formula increased the body weight of the rats with IDA more effectively. Similar results were obtained by Marciano *et al.*,^49^ who studied the effect of inulin and oligosaccharides on IDA and reported that the two prebiotics had the same gain weight as that of the non-anemic animals. Also, it has been reported that feeding oligosaccharides can enhance the animal growth by promoting the immune functions.^50,51^

**Table 2:** Body weight gain and organs weight and coefficient^a^ of control and anemic rates fed for 4 weeks with yoghurt fortified with iron and short- or long-chain inulin. Data are the means ± standard deviations

Animal groups^b^	Weight in gram				
	Body weight gain	Heart	Liver	Kidney	Spleen
G1	18.33 ± 1.52^b^	0.53 ± 0.01^c^	4.32 ± 0.08^d^	0.94 ± 0.10^d^	0.32 ± 0.03^d^
G2	5.33 ± 0.52^e^	0.77 ± 0.09^a^	6.11 ± 0.45^a^	1.39 ± 0.13^a^	0.63 ± 0.04^a^
G3	7.33 ± 1.52^d^	0.63 ± 0.05^b^	5.46 ± 0.28^c^	1.09 ± 0.11^c^	0.48 ± 0.05^c^
G4	17.33 ± 2.08^c^	0.61 ± 0.08^b^	5.90 ± 0.38^b^	1.31 ± 0.11^a,b^	0.59 ± 0.11^b^
G5	19.67 ± 0.06^a^	0.69 ± 0.03^b^	5.51 ± 0.15^c^	1.29 ± 0.12^b^	0.50 ± 0.13^b,c^

**Organ coefficient (%)**					
G1	—	2.89 ± 0.06^c^	23.56 ± 1.98^e^	5.12 ± 0.75^d^	1.73 ± 0.03^d^
G2	—	5.00 ± 0.08^a^	39.85 ± 2.40^a^	9.00 ± 1.78^a^	4.00 ± 0.98^a^
G3	—	3.63 ± 0.10^b^	31.50 ± 2.89^c^	6.28 ± 1.01^c^	2.76 ± 0.45^c^
G4	—	3.52 ± 0.12^b^	34.00 ± 2.00^b^	7.55 ± 1.12^b^	3.40 ± 0.85^b^
G5	—	3.50 ± 0.15^b^	28.00 ± 1.67^d^	6.55 ± 0.70^c^	2.50 ± 0.09^c^

a Organ coefficient (g/100g) = [organ weight (g)/rat body weight (g)] × 100.

b G1: non anaemic animals feed with standard diet mixed with (1 : 1) inulin-free yoghurt containing 90 mg kg^−1^ Fe_2_(SO_4_)_3_, G2: anaemic animals feed with standard diet mixed with (1 : 1) inulin-free yoghurt containing 10 mg kg^−1^ Fe_2_(SO_4_)_3_, G3: anaemic animals feed with standard diet mixed with (1 : 1) inulin-free yoghurt containing 90 mg kg^−1^ Fe_2_(SO_4_)_3_, G4: anaemic animals feed with standard diet mixed with (1 : 1) yoghurt containing 90 mg kg^−1^ Fe_2_(SO_4_)_3_ and 4% long chain inulin and G5: anaemic animals feed with standard diet mixed with (1 : 1) yoghurt containing 90 mg kg^−1^ Fe_2_(SO_4_)_3_ and 4% short chain inulin. Superscript letters in the same column denote significant differences (*P* ≤ 0.05).

### 3.2 Organ weights and coefficients

The organ weights and organ coefficients are a basic part of the determination of drug safety and are used to estimate deep internal organ disease.^52^ The organ weights and coefficients of the anemic rats subjected to different groups are presented in Table 2. The heart weight in the anemic animals was significantly higher than that in the normal control group (*P* ≤ 0.05), which indicated the initial symptoms of cardiac hypertrophy due to iron deficiency.^53^ The heart coefficients in all the animals subjected to G3, G4, and G5 were significantly reduced (*P* ≤ 0.05) and yogurt fortified with iron, depending on the presence of inulin and the length of its chain, restored the heart coefficient close to the normal level. The most restored effect was associated with G4 animals, which were fed long inulin-containing yogurt fortified with iron.

A significant increase in the liver weight and coefficient was observed in the anemic animals, indicating that iron deficiency might cause an increase in the liver volume due to inflammatory conditions, as observed during the histological examination of liver tissues of animals subjected to the treatment groups G3, G4, and G5. After 4 weeks of feeding yogurt fortified with iron, the liver coefficients in G3, G4, and G5 significantly decreased (*P* ≤ 0.05) compared with the low-iron fed animals (G2). These results were contradictory to those reported by He *et al.*^46^ The spleen weights and coefficients were significantly (*P* ≤ 0.05) higher in anemic animals compared with those of the control animals (G1). Spleen hypertrophy was observed in anemic rats (G2), which may be attributed to the high rate of cell proliferation caused by iron deficiency.^54^ Rats subjected to groups G3, G4, and G5 showed a slight reversal in the spleen hypertrophy and were still far from the normal status. The kidney coefficients of the anemic animals (G2 to G5) were significantly (*P* ≤ 0.05) higher compared with those in the normal control group (G1). Also, swelling of the kidney was clearly observed in the anemic animals, which was described as edema and attributed to the deficiency of iron.^46,55^ However, the coefficients of the kidney in all the formula supplementation groups exhibited a moderate reduction in comparison with those in the normal control group (*P* ≥ 0.05), which were still far from that of the control. Inulin-containing yogurt fortified with iron could restore the statuses of all the organs and had a particular advantage for the heart and the kidneys.

### 3.3 Hematological parameters

Traditionally, blood tests are successfully applied to detect IDA.^56^ Among blood components, the concentration of blood hemoglobin (Hb), the major red blood cell component that transports oxygen to different body tissues and organs, is directly correlated with the anemic status of the human body.^57^ At the end of the experiment, the Hb content (9.70 ± 0.30 g L^−1^) in the anemic animals (G2) was significantly lower (*P* ≤ 0.05) than that (13.50 ± 0.30 g L^−1^) in the normal animals (G1), as shown in Table 3. This confirmed that the anemic status (induced by the peritoneal injection of phenylhydrazine) continued throughout the experimental duration. In general, iron deficiency is due to the relief of the circulation of feasible iron in the blood and consequently, the reduction of the Hb content.^58,59^

**Table 3:** Blood parameters of control and anemic rates fed for 4 weeks with yoghurt fortified with iron and short- or long-chain inulin. Data are the means ± standard deviations

Animal groups^a^	Hematological parameters^b^								
	WBC thousand cell per μL	LY%	MO%	GR%	RBC million cell per μL	Hgb g dL^−1^	HCT%	RDW%	PDW f L^−1^
G1	8.50 ± 1.00^b^	72.80 ± 0.80^a^	9.90 ± 0.10^c^	18.10 ± 0.50^e^	6.10 ± 0.20^a^	13.50 ± 0.30^a^	40.10 ± 0.9^a^	20.30 ± 0.20^d^	7.70 ± 0.20^c^
G2	9.30 ± 0.30^a^	56.70 ± 0.80^d^	11.80 ± 0.60^a^	28.50 ± 0.60^a^	5.20 ± 0.30^c^	9.70 ± 0.30^d^	37.90 ± 1.1^c^	33.80 ± 0.30^a^	8.60 ± 0.00^a^
G3	5.10 ± 0.10^c^	72.10 ± 0.10^b^	9.90 ± 0.10^c^	21.80 ± 0.90^d^	5.30 ± 0.10^c^	12.00 ± 0.20^b,c^	38.30 ± 0.6^b^	23.80 ± 1.60^c^	8.40 ± 0.10^a,b^
G4	4.40 ± 0.60^d^	64.50 ± 2.00^c^	9.30 ± 1.30^d^	27.50 ± 1.00^b^	5.60 ± 0.10^b^	12.70 ± 0.50^a,b^	39.50 ± 1.80^a^	29.70 ± 0.60^b^	7.90 ± 0.60^b,c^
G5	5.30 ± 0.30^c^	64.01 ± 2.40^c^	10.70 ± 0.40^b^	24.00 ± 0.90^c^	5.50 ± 0.20^b^	11.56 ± 1.80^c^	39.70 ± 0.60^a^	28.80 ± 0.40^b^	7.10 ± 1.00^d^

a See foot note Table 2.

b WBC: white blood cells, LY: lymphocytes, MO: monocytes, GR: granulocytes, RBC: red blood cells, Hgb: hemoglobin, HCT: hematocrit, RDW: red blood volume and PDW: platelet distribution width. Superscript letters in the same column denote significant differences (*P* ≤ 0.05).

The Hb contents in animals subjected to groups (G3, G4, and G5) were significantly higher than that of the control anemic group (G2). In general, Hb concentration among experimental groups was in the order G1 > G4 > G3 > G5 > G2 The Hb contents in the animals belonging to G4, which were fed yogurt containing long-chain inulin, were slightly lower but insignificant (*P* ≥ 0.05) compared with those found in the control healthy animals (G1). The same effect was observed by Marciano *et al.*,^49^ who reported that feeding anemic animals with HP inulin or oligosaccharides elevates the Hb level up to the control of normal animals. Therefore, the presented results confirmed that our formula fortified by iron and supplemented with inulin could significantly increase the blood Hb content, in particular, long-chain inulin that had a good recovery effect on the Hb content. Zhang *et al.*^60^ confirmed that anemic rats fed the low-iron diet showed a mild increase (*P* ≤ 0.05) in the hemoglobin level after 21 days of fructooligosaccharide (FOS) supplementation when compared to rats without FOS.

Red blood cells (RBC), which restrain 90% Hb, are the most abundant cells suspended in the blood. They play a critical function in gas transportation into the body and an adequate number of RBCs is very important to preserve the acid–base balance of the body at the normal status.^61^ The results in Table 3 indicate that anemic rats (G2) had the lowest count of RBCs. Similarly, Moreno-Fernandez *et al.*^62^ reported that the induction of anemia in rats was correlated with a significant decrease in the RBC count. Feeding yogurt-containing inulin led to a significant increase in the count of RBC. The counts of RBC in animals assigned to group G4 were significantly (*P* ≤ 0.05) different from those of the control healthy group (G1).

Hematocrit (HCT) is known as the ratio of the RBC volume to the whole blood volume.^57^Table 3 shows that the RBC count and HCT values in the anemic animals assigned to G2 were significantly lower than those in the control group G1 (*P* ≤ 0.05). This is an original sign because RBC and HCT are positively remedial with Hb.^57,63^ Iron deficiency led to a decrease in the Hb content (Table 3), which caused a decline in the numbers of RBCs and HCT values in rats with IDA. In contrast, the RBC counts and HCT values in the three iron-supplemented groups were significantly higher (*P* ≤ 0.05) compared with the anemic group. Also, the RBC counts and HCT values in the inulin-containing yogurt fortified with ferric sulfate diets groups increased to normal levels (*P* ≤ 0.05). These results indicated that the formula inulin-containing yogurt fortified with iron was sufficient to increase the RBC count and HCT value in rats with anemia to normal levels.

### 3.4 Enzymatic biomarkers for liver activity

Alkaline transaminase (ALT) is a transaminase enzyme, which is necessary for different body tissues. ALT is mostly found in the liver. It plays a crucial role in the Cahill cycle and is clinically used as a biomarker in health monitoring and liver function.^9^ Increasing the ALT level may indicate hepatocellular injury, though etiologies diverge from rudimentary liver disorders to infections, malignancy, and heart failure.^64^ Comparable with ALT, aspartate transaminase (AST) is another transaminase utilized as a biomarker for liver health conditions. It differs from ALT in that AST is found more in tissues outside the liver, making it a minus specific biomarker for hepatocellular damage.^64^

The anemic control group is characterized by significant (*P* ≤ 0.05) elevated levels of ALT and AST and low levels of albumins that indicate a liver hepatocellular injury; this finding is correlated with the study of He *et al.*^46^ and Wang *et al.*^48^ All the iron-supplemented animals exhibited no differences (Table 4); they showed low ALT, ASP, and high albumin content in comparison to the anemic control group but not equal to the normal group. These results indicate an enhancement in the liver functions in animals fed with the three supplemented formulas, while the most improvement, especially an increment in the AST content to normal levels, was observed with G4, which was fed-yogurt fortified with iron and supplemented with long-chain inulin. Normally, an affirmative relation is found between the albumin level and the amount of iron stored in the body.^65^

**Table 4:** Serum levels of liver function enzymes, antioxidant and calcium and iron of control and anemic rates fed for 4 weeks with yoghurt fortified with iron and short- or long-chain inulin. Data are the means ± standard deviations

Animal groups^a^	Experimental tests							
	ALT^b^ U L^−1^	AST^c^ U L^−1^	ALP^d^ U L^−1^	TP^e^ g dL^−1^	Albumin g dL^−1^	Globulin g dL^−1^	Calcium mg dL^−1^	Fe μg dL^−1^
G1	12.72 ± 0.22^d^	17.04 ± 0.03^d^	178.80 ± 8.62^e^	7.51 ± 0.50^a^	4.69 ± 0.08^a^	4.12 ± 0.67^a^	11.08 ± 0.35^a^	141.85 ± 5.44^a^
G2	39.08 ± 0.68^a^	38.32 ± 0.67^a^	501.50 ± 2.12^a^	6.60 ± 0.01^b^	2.67 ± 0.06^d^	3.93 ± 0.08^b^	8.00 ± 0.38^c^	45.65 ± 3.88^e^
G3	27.04 ± 0.79^b^	30.92 ± 1.45^b^	355.00 ± 5.65^b^	6.86 ± 0.18^b^	3.34 ± 0.25^b^	3.52 ± 0.07^c^	11.28 ± 1.30^a^	81.95 ± 3.46^d^
G4	22.09 ± 0.74^c^	18.26 ± 0.73^c,d^	287.90 ± 5.94^d^	7.42 ± 0.10^a^	3.47 ± 0.02^b^	3.95 ± 0.12^b^	11.00 ± 0.00^a^	126.35 ± 5.44^b^
G5	22.80 ± 1.07^c^	19.45 ± 0.71^c^	325.65 ± 11.38^c^	7.37 ± 0.26^a^	3.11 ± 0.06^c^	4.26 ± 0.325^a^	10.94 ± 1.01^b^	115.75 ± 3.32^c^

a See foot note Table 2.

b Alanine aminotransferase.

c Aspartate aminotransferase.

d Alkaline phosphatase.

e Total protein. Superscript letters in the same column denote significant differences (*P* ≤ 0.05).

### 3.5 Serum iron (SI) content

The bioavailability of iron can be determined by measuring the concentration of serum iron (SI).^65^ The SI concentration of animals fed formula supplementation gradually improved over the anemic animals. The most improvement was observed within animals fed two types of inulin (Table 4). Hence, the present results further indicate that the long inulin supplemented formula more effectively increased the bioavailability of iron. The same effect on the SI level was observed by beta-glucans, mannan oligosaccharides, and chitooligosaccharides.^50,51^ Moreover, long inulin contributed to an increase in the level of serum calcium of the anemic animals up to the normal level of non-anemic group; this effect was less pronounced with short-chain inulin supplementation (Table 4).

This effect may be attributed to the upregulation of genes related to Fe metabolism, especially the metal transporter 1 and ferroportin genes, regardless of the chain length.^66^ On the other hand, our study suggested that the chain length of oligosaccharides has an effective role since the long-chain inulin presented a promising level of SI over the short-chain inulin. Also, Marciano *et al.*^49^ confirmed a high expression level of divalent metal transporter 1 (DMT1) protein in the duodenum, caecum, and colon cells, which indicated that a high level of iron bioavailability was associated in animals fed HP inulin over those fed the oligosaccharides (10 unites fructose) and the control normal animals. Another hypothesis is that prebiotics promote iron absorption *via* prebiotic fermentation by beneficial microorganisms present in the colon, which produce short-chain fatty acids (SCFAs). The SCFAs may contribute to decreasing the pH of the luminal content, increased iron solubility by enhancing the reduction of Fe(iii) to Fe(ii), and stimulate the proliferation of epithelial cells, thus expanding the absorptive surface area.^67^ However, the available research about the effect of the inulin properties, especially the polymerization degree on iron absorption, is inconsistent.

### 3.6 Antioxidant activities in the serum and liver

The antioxidant enzyme system for the body is fundamentally composed of superoxide dismutase (SOD), catalase (CAT), and plasma glutathione peroxidase (GSH-PX). SOD is the premier line of defense against the body's reactive oxygen species (ROS), CAT is an opener enzyme in the normal defense system, and GSH-PX has an important protective role against lipid membrane oxidation.^68^ SOD catalyzes the disproportionation of superoxide radicals to hydrogen peroxide (H_2_O_2_), whereas CAT and GSH-PX convert H_2_O_2_ to H_2_O, thereby protecting the cells from damage by ROS.^6,9^

The results of the antioxidant activities in the serum and liver are shown in Table 5. In comparison with the normal control group, the activities of SOD and GSH-PX were significantly lower (*P* ≤ 0.05) and the thiobarbituric acid-reactive substance (TBARS) content was significantly higher (*P* ≤ 0.05) in the serum and liver homogenate. This might indicate that IDA resulted in the accumulation of ROS and caused oxidative stress (OS) by disrupting the balance of the antioxidant enzyme system.^69,70^

**Table 5:** Antioxidants level of liver tissue for control and anemic rates fed for 4 weeks with yoghurt fortified with iron and short- or long-chain inulin. Data are the means ± standard deviations

Animal groups^a^	Biochemical parameters			
	TP^c^ g dL^−1^	TBARS^b^ μmol g^−1^ tissue	GPx^d^ U per mg protein	SOD^e^ U per mg protein
G1	7.11 ± 0.00^a^	1.41 ± 0.54^d^	0.35 ± 0.00^a^	2.21 ± 0.01^a^
G2	6.05 ± 0.10^c^	8.59 ± 0.18^a^	0.18 ± 0.01^d^	1.29 ± 0.00^d^
G3	6.66 ± 0.16^b^	5.41 ± 0.72^b^	0.22 ± 0.02^c^	1.79 ± 0.06^c^
G4	7.37 ± 0.08^a^	4.10 ± 0.00^c^	0.28 ± 0.00^b^	2.05 ± 0.20^b^
G5	6.72 ± 0.08^b^	4.91 ± 0.16^b,c^	0.25 ± 0.01^b,c^	1.88 ± 0.12^c^

a See foot note Table 2.

b Thiobarbituric acid-reactive substance.

c Total protein.

d Glutathione peroxidase.

e Superoxide dismutase. Superscript letters in the same column denote significant differences (*P* ≤ 0.05).

The activities of SOD and GSH-PX significantly increased (*P* ≤ 0.05) and the TBARS content was significantly reduced (*P* ≤ 0.05) in the G3, G4, and G5 groups compared with the anemic group (G2), as shown in Table 5. On the other hand, there was no significant (*P* ≥ 0.05) differences in the activities of SOD and GSH-PX, and the TBARS content in the sera of animals fed yogurt-containing either long-(G4) or short-(G5) chain inulin. Indeed, the restoration of the antioxidant activity in the livers of anemic animals to the normal status was more evident in animals fed yogurt-containing long-chain inulin (G4), followed by those belonging to G5 and G3, respectively. This finding might indicate that long-chain inulin-containing yogurt could alleviate the damage to the hepatocytes due to IDA and lipid peroxidation and reduce the destructive effect of oxidative stress in the body.^70^ Similarly, a previous study confirmed the positive relationship between polysaccharides and antioxidant activity in anemic rats.^71^ The presented study confirmed that the changes in the antioxidant activity in the liver and serum (Tables 4 and 5) in the anemic groups were consistent with the SI content, indicating that iron supplementation can improve liver function and its antioxidant capacity.

### 3.7 Histological analysis of the liver

**Fig. 1:**
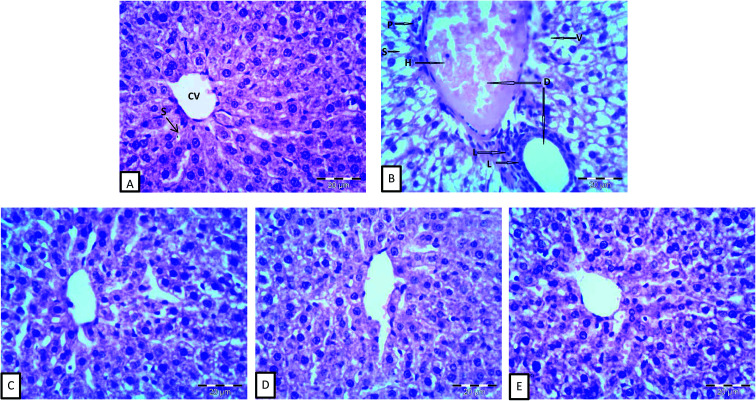
Effect of iron and inulin supplementation on histomorphological changes in the liver sections of rats: (A) control group; showing normal hepatocytes architecture central vein (CV) and normal blood sinusoids (S). (B) Phenyl hydrazine-treated rates showing vacuolization (V) and hemorrhage (H) in the central, aggregation of lymphocytes (L), and loss of the normal architecture, degenerated hepatocytes with pyknotic nuclei (P). (C), (D) and (E) phenyl hydrazine induction and feed fermented milks fortified with iron, iron and high density inulin and iron and low density inulin respectively, showed that most of the histological alterations induced after the phenyl hydrazine induction were markedly reduced. (H & E; ×400).

The histomorphological structures of the liver tissues are shown in Fig. 1. Normal cellular architecture with distinct hepatic cells, sinusoidal spaces, and a central vein was observed in the control of normal animals (G1), as shown in Fig. 1A. On the other hand, the histopathological observations in Fig. 1B showed histopathological alterations in the liver, dilatation of both central and portal veins, distension and hemorrhage in the central vein, loss of the normal hepatocytes architecture, degenerated hepatocytes with pyknotic nuclei, hepatocyte vacuolization, dilation and activation of Kupffer cells of hepatic sinusoids, and the aggregation of lymphocytes. Similar results for normal and anemic livers were reported by He *et al.*^46^ The differences in hepatic sinusoids and hepatic plates between anemic and normal livers, observed in the present study, might be attributed to the inhibition of DNA synthesis in the hepatocytes of anemic animals.^46,72^ Moreover, iron deficiency has been reported to cause the disorder of lipid metabolism in the liver, which caused the appearance of fat vacuoles in the hepatocytes,^73^ as shown in anemic livers.

Liver cells from G3, which were fed inulin free-yogurt, showed that the hepatocytes were partially complete, the nuclei were large and roundish, and there were hardly any fat vacuoles in the hepatocytes; however, the liver plates were slightly dilated (Fig. 1C). The hepatocytes in the liver sections of animals subjected to G4 and G5 treatment, which were fed yogurt supplemented with iron and fortified with long or low chain inulin, respectively, were complete and appeared to be close to the structure of the hepatocytes of normal animals (Fig. 1D and E). The boundaries of the liver plates of both the groups were clear, the hepatic sinusoids were ordered, and there was a small number of fat vacuoles in the hepatocytes. These results indicated that the livers of the rats belonging to groups G4 and G5 were gradually reversed to the normal structure. In general, the disorder in the arrangement of hepatic sinusoid and plates and the presence of fat vacuoles in the hepatocytes, which were induced by the anemia status, were improved to different levels, depending on the type of yogurt used. The photomicrographs of the liver cells for the liver sections of G4 and G5 were similar to those for the normal group. Importantly, the effect of inulin-containing yogurt in repairing the liver tissue was better than those of inulin-free yogurt.

## 4 Conclusion

Yogurt fortification with Fe_2_(SO_4_)_3_ and inulin supplementation could be a promising approach to improve the ability of yogurt to deliver iron metal into the body and enhance its bioavailability. The results presented in this study indicated that long- or short-chain inulin could significantly improve the anemic status caused by iron deficiency. Yogurt containing long-chain inulin appeared to be more effective than that containing short-chain inulin. This formula of yogurt, which was fortified with Fe_2_(SO_4_)_3_ and long-chain inulin, improved the bioavailability of iron as well as the liver function and the antioxidant capacity. This formula has the potential to be used as the basis for the development of a new dairy functional product or as an iron supplement.

## Conflicts of interest

The authors declare that they have no conflicts of interest.

## Supplementary Material
